# Association between physical activity education and prescription during prenatal care and maternal and fetal health outcomes: a quasi-experimental study

**DOI:** 10.1186/s12884-023-05808-x

**Published:** 2023-07-05

**Authors:** Latifa Saidi, Pierre D. Godbout, Camille Morais-Savoie, Pierre Philippe Wilson Registe, Mathieu Bélanger

**Affiliations:** 1 Faculty of Medicine and Health Sciences, Université de Sherbrooke, 2500, boul. de l ’Université, Sherbrooke, Québec J1K 2R1 Canada; 2 School of Nursing, Université de Moncton, Campus de Shippagan, 725, Rue du Collège, Bathurst, NB E2A 3Z2 Canada; 3 Centre de Formation Médicale du Nouveau-Brunswick, Université de Sherbrooke, Pavillon J-Raymond-Frenette, 50 Rue de La Francophonie St, Moncton, NB E1A 7R1 Canada; 4 Department of Family and Emergency Medicine, Université de Sherbrooke, Centre de Formation Médicale du Nouveau-Brunswick, Vitalité Health Network, Pavillon J-Raymond-Frenette, 18, Avenue Antonine-Maillet, Moncton, NB E1A 3E9 Canada

**Keywords:** Pregnancy, Physical activity, Physical activity promotion, Gestational weight gain (GWG), Gestational diabetes (GD), Gestational hypertension (GHT), Fetal macrosomia

## Abstract

**Background:**

Physical activity (PA) during pregnancy is associated with healthy gestational weight gain (GWG) and a reduced risk of developing gestational diabetes (GD), gestational hypertension (GHT) and fetal macrosomia. However, in Canada, less than 20% of pregnant women meet PA recommendations. This study assessed associations between an intervention including PA education by prenatal nurses and a PA prescription delivered by physicians and fetal and maternal outcomes.

**Methods:**

This is a quasi-experimental study. Two groups of women who received their prenatal care at the obstetrics clinic of a university hospital were created. In the first group, 394 pregnant women followed at the clinic received standard care. In the second group, 422 women followed at the clinic received standard care supplemented with education on the relevance of PA during pregnancy and a prescription for PA. Data for both study groups were obtained from the medical records of the mothers and their newborns. Logistic regressions were used to compare the odds of developing excessive GWG, GD, GHT, and fetal macrosomia between the two study groups.

**Results:**

The addition of PA education and PA prescription to prenatal care was associated with 29% lower odds of developing excessive GWG (adjusted odds ratios (OR) 0.71, 95% confidence intervals (CI) 0.51–0.99), 73% lower odds of developing GHT (0.27, 0.14–0.53), 44% lower odds of fetal macrosomia (> 4 kg) (0.56, 0.34–0.93), and 40% lower odds of being large for gestational age (0.60, 0.36–0.99). The intervention was not associated with a difference in odds of developing GD (0.48, 0.12–1.94).

**Conclusions:**

The inclusion of education and prescription of PA as part of routine prenatal care was associated with improvements in maternal and fetal health outcomes, including significantly lower odds of GWG, GHT and macrosomia.

## Background

It is recommended that pregnant women participate in 150 min of PA per week to prevent excessive gestational weight gain (GWG) [1, 2], gestational diabetes (GD), gestational hypertension (GHT) and fetal macrosomia [1, 3]. Nevertheless, in Canada, fewer than 20% of pregnant women adhere to this recommendation [4]. Moreover, approximately 50% of pregnant women in Canada gain more than the recommended weight during their pregnancy [5], a percentage similar to other developed countries [6]. Excessive GWG is associated with an increase incidence of maternal and neonatal complications [6, 7]. For the mother, excess GWG increases the risk *of premature birth *[8]* and obesity *[7]*, having a C-Sect. *[9]*, and developing GD *[10]*, GHT *[11]*, and excess weight *[7]*.* In neonates, excessive GWG increases the incidence of macrosomia [8, 10] and stillbirth [10]. The incidence of GD [12], GHT [13] and macrosomia [14] has been steadily increasing over the past 20 years. In Canada, about 10% of pregnant women have DG [15], GHT [13] and macrosomia [14]. Undiagnosed or untreated GD increases the risk of maternal and perinatal morbidity [16, 17]. For the mother, GD increases the risk of GHT [16], preeclampsia [18], excess maternal weight [19], premature birth, having a C-section, developing GD in future pregnancies [16], and type 2 diabetes, whether in the years following delivery [16, 20] or later in life [16, 18]. For the infant, GD increases the risk of macrosomia and fetal malformations [17], birth trauma [20], obesity and childhood diabetes [16], glucose intolerance in early adulthood [21], and stillbirth [18]. GHT is the leading cause of maternal and fetal morbidity and mortality [22–24]. GHT increases the risk of chronic high blood pressure, cardiovascular events [23, 25], and diabetes [24]. Severe arterial hypertension during pregnancy can cause complications, such as heart or kidney failure, hypertensive encephalopathy, aortic dissection, and stroke [25]. For the fetus, GHT can lead to intrauterine growth restriction, low birth weight, and premature birth [24].

Macrosomia is associated with poorer health status throughout life [26]. It increases the incidence of fetal and maternal mortality and morbidity [27]. For the mother, it can result in difficult and occasionally traumatic vaginal delivery [28] or an emergency C-Sect. [18, 27, 29]. Macrosomic newborns are at increased risk of excess weight and obesity in childhood and adulthood [30], hypertension, adult ischemic heart disease, type 2 diabetes, and cancer in childhood and adulthood [31].

Several studies have reported that individualized and personalized interventions appear to be effective in promoting PA [32–35]. These interventions include information sharing [35], counseling [32, 34] and individual education [33]. Interventions that appear to be particularly effective involve education provided by nurses [33] as well as counseling provided by physicians [34]. Personalized education allows information to be tailored to the needs of each individual, thereby increasing knowledge and enhancing PA-related motivation [33]. Physician advice is also highly respected among patients and can positively contribute to changing their PA levels [36–38]. Randomized controlled trials [39, 40] and systematic reviews [41, 42] have shown that prescribing PA is associated with a significant increase in PA behaviors, even in patients who were initially sedentary [39, 40] or who initially did not intend on changing their behaviors [39]. However, the effectiveness of PA prescriptions during prenatal care has not yet been assessed.

The transtheoretical model suggests tailoring behavioral interventions to each individual’s stage of behavior change [43, 44]. According to this model, individuals may progress through five stages of behavior change: precontemplation, contemplation, preparation, action, and maintenance. Tailored interventions for each stage increase the likelihood of perceiving changes in PA as something positive [45–48].

Despite evidence supporting the promotion of prenatal PA as beneficial to the health of both mother and child, the most effective means of preventing certain maternal and fetal outcomes is still unclear [49]. Combining education and the prescription of personalized PA according to stage of behavior change does seem promising in terms of preventing GWG, GD, GHT, and macrosomia. The objective of this quasi-experimental study is to compare fetal and maternal health outcomes among women who received standard prenatal care and others who additionally received a simple intervention that can be integrated into the regular setting of a pregnancy follow-up clinic and which combines structured PA education offered by a nurse with a personalized PA prescription delivered by a physician. The primary outcome is the prevalence of excessive GWG, while the secondary outcomes are the prevalence of GD, GHT, and fetal macrosomia. More specifically, the study sets out to test the following hypotheses: in comparison to pregnant women who received standard care, those who also received structured education and a PA prescription are more likely to complete their pregnancies with an optimal GWG and a lower incidence of GD and GHT, and to deliver infants with a healthy birth weight.

## Methodology

### Study design

This quasi-experimental study was conducted under regular practice conditions of an obstetric clinic. In terms of methodological approach, we used patient records data to follow up and compare the maternal and fetal outcomes of two distinct groups of pregnant women: those who frequented the obstetric clinic when the standard program of care was being offered, and those who were exposed to an enhanced program of care at the clinic, which included PA education and prescription of PA during pregnancy. The study was approved by the Research Ethics Board of the Vitalité Health Network (Bathurst, New Brunswick, Canada), which oversees the health institution where the study was conducted, and by the Research Ethics Board of the Université de Moncton (Moncton, New Brunswick, Canada).

### Participants

Eligible participants were pregnant women who had a follow-up at the Obstetric Clinic of the Dr. Georges-L.-Dumont University Hospital Centre (DGLDUHC), located in Moncton, New Brunswick, Canada. At this clinic, the first usual visit for all women takes place at approximately week 12 of pregnancy. Subsequent appointments follow a clearly defined schedule. Thus, pregnant women return to the clinic every four weeks until they reach 32 weeks of gestation, then every two weeks until the 36–week mark, and then every week until delivery. Pregnant women are monitored by three categories of health professionals: nurses who specialize in prenatal care, delivery physicians, and obstetrician-gynecologists. To be recruited as a study participant, women were required to have received care at the obstetric clinic before week 16 of gestation, understand and speak either English or French, be at least 18 years of age, and be pregnant with a singleton. Excluded from the study were women with eating disorders, pregnancy-related complications, or general medical conditions not associated with pregnancy (all assessed and noted in medical files by physicians as part of routine care) and which required specialized maternity care and included contraindication for physical activity (i.e., anorexia, lumbar hernia, deep vein thrombosis, and placental anomalies). The same inclusion and exclusion criteria were applied for both study groups.

All women whose pregnancy follow-up began after April 2019 were exposed to the enhanced clinical care services. Within 48 h of delivery, these women were asked to provide written informed consent so that the research team could review their medical records and those of their newborns, thus collecting data relevant to this study. For the comparison group, the research team considered the medical records of all women who were followed at the obstetric clinic and gave birth at least 6 months preceding the offer of enhanced care. This period was selected to avoid contamination between groups as we started discussing the potential benefits of the enhanced intervention in the months leading to its implementation. Given the challenges associated with retracing patients discharged from the hospital months ago, the ethics committees granted us an exemption to seek consent from this historical comparison group.

### Intervention

For the enhanced clinical care services, nurses were trained to offer standardized PA education to pregnant women. Specifically, a one-hour training session aimed to standardize how nurses provided care and education to pregnant women. At this session, nurses received an information package detailing the research project, an educational brochure about PA for pregnant women, the weight gain tracking chart and the transtheoretical model's stages of PA behaviour change scale. Nurses were taught how to use the information tools to deliver education to pregnant women on current PA guidelines, the benefits of PA, recommendations related to GWG, and the consequences of excessive GWG, GD, GHT, and fetal macrosomia on maternal and fetal health. Thereafter, nurses delivered the educational information to pregnant women during their first prenatal visit at the clinic. Pregnant women received an explanatory brochure with infographics to support the education on PA. During this first visit, pregnant women were asked to identify their stage of behavior change according to the transtheoretical model [50]. The first prenatal visit also involved a discussion on individual weight gain recommendations based on body mass index (BMI) recommendations issued by the Institute of Medicine (IOM). Individual weight gain was monitored by nurses during each subsequent visit until week 37 of gestation. For individualized follow-up of GWG, and to help guide participants in monitoring their own GWG, weight gain information was captured on a personalized chart with marked intervals highlighting recommended weight [51]. Following the education session with the nurse, participants received a personalized PA prescription written by a physician. Physicians were previously trained in PA prescribing using the model developed by Exercise is Medicine Canada (EMC) [52]. This one-hour training was offered by an EMC trainer. Physicians were provided with an information package detailing the research project, an educational brochure about PA for pregnant women, the weight gain tracking chart, the transtheoretical model's stages of PA behaviour change scale as well as a standardized prescription pad for PA. The training included a review of the evidence supporting PA prescribing, presentation of tools for prescribing PA, and practical exercises including presentation of strategies to support writing PA prescriptions for women at each stage of behaviour change.

### Standard practice

During their first visit to the obstetric clinic, women in the standard practice group received approximately 1-min of general information about PA and GWG during pregnancy from nurses. For women in the standard practice group, there was no monitoring of GWG using the personalized weight gain chart with recommended weight ranges. As part of standard practice, women in both study groups received written and oral information about dietary recommendations for pregnant women as outlined in Canada’s Food Guide [53] and in *Healthy Pregnancy… Healthy Baby – A New Life*, an online prenatal guide published by the Government of New Brunswick [54]. Among other things, the nurses stressed the importance of regular meals, snacks, multivitamins, foods that contain iron, folic acid, omega-3 fats, and fiber. Nurses also discussed foods to limit, such as low-nutrient foods, fried foods, artificial sweeteners, and caffeine. For both groups, women experiencing excessive GWG could be referred to a nutritionist, but access to this service was not documented.

### Maternal and fetal outcomes

All data were obtained retrospectively from the medical records of the mothers and their newborns. Socio-demographic data and data on each of the study outcomes were routinely collected by nurses in the obstetric clinic. Pre-pregnancy weight was self-reported by the pregnant woman during her first clinic appointment. Thereafter, the woman’s weight was taken and recorded in her obstetrical record during each clinic appointment throughout the pregnancy by the nursing staff. All women were weighed using the same electronic scale which was calibrated and validated by a medical engineering department according to the manufacturer’s schedule. Total GWG was obtained by calculating the difference between weight recorded at the 37-week mark and maternal weight measured during the first routine prenatal visit. The final weight measurement considered for all mothers was assessed at week 37 of gestation since, later in pregnancy, weight can be affected by swelling [55]. The appropriate total GWG for a normal, singleton pregnancy was based on IOM recommendations [56]. The Canadian GWG classification system depends on the mother’s pre-pregnancy BMI [51]. BMI was based on self-reported height and retrospective weight. The IOM has issued the following GWG recommendations: 12.5 to 18 kg, for women who are underweight; 11.5 to 16 kg, for women who are of normal weight; 7 to 11.5 kg, for women who are overweight; and 5 to 9 kg, for women who are obese [51].

GD was diagnosed according to guidelines issued by the Society of Obstetricians and Gynaecologists of Canada and the Canadian Diabetes Association [16, 17]. In the absence of high-risk factors for GD (≥ 35 years of age, high-risk ethnicity (Native American, African, Asian, Hispanic, South Asian), corticosteroid use, obesity, prediabetes, history of GD or macrosomia, parent with type 2 diabetes, polycystic ovary syndrome or acanthosis nigricans), all women were screened for GD between 24 and 28 weeks of pregnancy by measuring blood glucose one hour after ingestion of a 50 g glucose load. When high-risk factors for GD were present, screening was performed during the first half of pregnancy and repeated between 24 and 28 weeks, if the results were normal. A diagnosis of GD was made when plasma glucose was greater than 11.1 mmol/L. In women with a 1-h plasma glucose between 7.8 and 11.0 mmol/L, a second induced hyperglycemia test was performed with the ingestion of 75 g of glucose. This test led to a GD diagnosis when plasma glucose one hour after ingestion was ≥ 10.6 mmol/L, or when glucose two hours after ingestion was ≥ 9.0 mmol/L, or if the fasting glucose was ≥ 5.3 mmol/L.

Blood pressure was measured manually by nurses during each prenatal visit. GHT was defined by a blood pressure that first presents during the second half of pregnancy (≥ 20 weeks). A diagnosis of GHT was established by a systolic blood pressure ≥ 140 mmHg and/or a diastolic blood pressure ≥ 90 mmHg [22, 57].

Adherence to prenatal care was measured as number of obstetric clinic visits during pregnancy. The weight of newborns was measured at birth, without clothing, using the Baby Weigh Scale by Medela Inc. (McHenry, Illinois), a standard digital scale. A medical engineering service calibrated and validated the scale at least once a year. Macrosomia was defined as a birth weight ≥ 4000 g [1, 28]. Newborns were considered as large for gestational age (LGA) if their birthweight for gestational age was above the 90^th^ percentile as determined using the method of Kramer et al. [58].

### Data analysis

Sample size was determined based on the hypothesis that the addition of education from a nurse and a PA prescription during pregnancy would be associated with a 10-point increase in the probability of having an optimal GWG according to IOM recommendations. Since approximately 33% of Canadian women gain the IOM-recommended weight [56], it was estimated that 369 participants per group would provide a power of 80% with a 5% alpha error probability of noting an increase in the proportion of women who will gain the recommended weight if it reaches 43% in the group exposed to an enhanced care offer. Comparison between groups was assessed using t-tests for continuous variables and chi-squares for categorical variables. The dependent variables GD, GHT, and fetal macrosomia were treated as dichotomous (yes or no) categorical variables. The dependent variable GWG was treated as an ordinal variable with three modalities (low, adequate, or high) [51]. GWG, blood pressure, and newborn weight were also treated as continuous variables. Blood pressure was assessed for all women with measures representing all of pregnancy as well as trimester-specific measures. Logistic regression models were used to compare the odds of GD, GHT, and fetal macrosomia among women in the two study groups. Similarly, polynomial logistic regression models were used to compare the odds of having any of the categories of GWG according to study group. Linear regression models were also used for GWG, blood pressure, and newborn weight, which were treated as continuous variables. Multivariate extensions of these regression models were used to adjust the results for potentially confounding variables (see tables for details). Multinomial regression was estimated for GWG coded as low, adequate and high with all participants. Sensitivity analyses were also conducted by repeating GWG analyses only with women who did not have GD. We also compared GWG between groups as z-scores as these are independent of gestational duration. For both groups, analyses related to GWG and macrosomia were restricted to participants with deliveries at term (≥ 37 weeks ± 3 days), but analyses related to LGA included all newborns, including premature babies. Analyses related to GD and GHT were restricted to women who did not have other types of diabetes or hypertension, respectively. Other outcomes, such as GWG by pre-pregnancy BMI categories, type of delivery, induction of labor, perineal tears, episiotomy, prematurity, shoulder dystocia, and Apgar at one and five minutes of life were also compared between groups using t-test or Chi-square statistics. Regression models for these variables were adjusted for BMI, age, and parity. Finally, in line with intent-to-treat analyses principles, data from all participants in the intervention group were included, regardless of whether they received the PA education and prescription of PA.

## Results

### Characteristics of participants

**Fig. 1:**
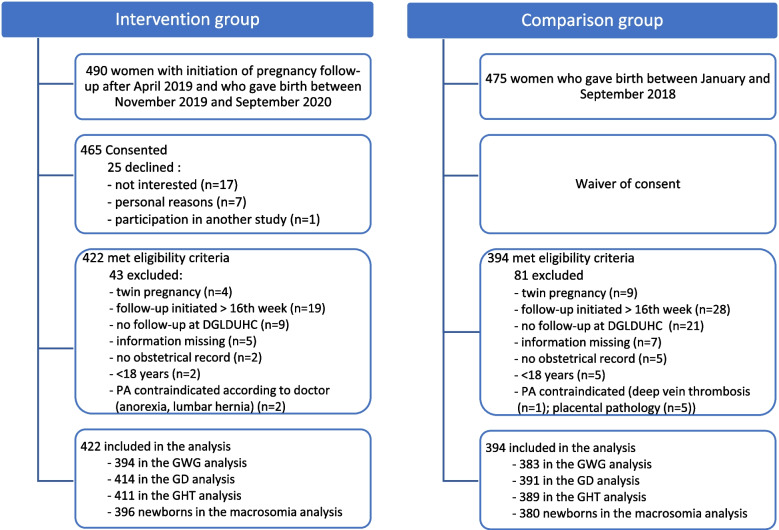
Flow chart of study participants. Abbreviations: DGLDUHC = Dr. Georges-L.-Dumont University Hospital Centre, PA = Physical activity, GWG = Gestational weight gain, GD = Gestational diabetes, GHT = Gestational hypertension

A total of 490 women were followed at the obstetric clinic during the period when prenatal care included PA education delivered by a nurse and a PA prescription. As a result, they were all invited to participate in the study following their delivery, which occurred between November 2019 and September 2020. Of these women, 465 (95%) consented to have their records and their newborn’s records accessed for this study, but 43 were eventually excluded, resulting in a total participant number of 422 for the intervention group (Fig. 1). Among this group, the GWG analyses were limited to the 394 women who delivered at ≥ 37 weeks (± 3 days). As for the GD analyses, they included the 414 women who did not have other types of diabetes. The 411 women who did not already have high blood pressure were included in the GHT analysis, and 396 newborns were included in the fetal macrosomia analyses.

For the comparison group, the medical records of 475 women who delivered between January and September 2018 were assessed for eligibility. Of these, 81 women were excluded (Fig. 1). As a result, the comparison group included 394 mother-infant pairs. A total of 383 women were included in the GWG analysis after exclusion of women with preterm delivery. The GD analysis involved 391 women who had no other types of diabetes. A total of 389 women did not already have high blood pressure and were thus included in the GHT analysis. Because of one stillbirth at 18 weeks of pregnancy, 380 newborns were included in the analysis of macrosomia at birth.

The group exposed to enhanced care and the group that received standard care did not differ with respect to women’s mean age, mean pre-pregnancy weight, or pre-pregnancy BMI (Table 1). BMI categories, marital status, education level, maternal ethnic background, gestational age at first visit, gestation duration, primiparity, and history of GD, GHT, depression or anxiety were also comparable between the two study groups. However, on average, women from the control group attended the obstetric clinic more frequently than those from intervention group during pregnancy (10.4 visits vs 9.6 visits, *p*-value < 0.001).

**Table 1:** Baseline characteristics of study participants

	**Intervention (*n* = 422)**	**Comparison group (*n* = 394)**	***p*-value**
Age (years)^a^	29.2 ± 5.3	29.3 ± 5.1	0.891
Height (cm)^a^	163.7 ± 7.1	163.6 ± 7.0	0.898
Weight before pregnancy (kg)^a^	71.5 ± 17.9	72.2 ± 19.7	0.609
BMI (kg/m^2^)^a^	26.7 ± 6.4	26.9 ± 7.0	0.586
BMI categories (n (%))			0.743
< 18.5 kg/m^2^	19/420 (4.5%)	14/393 (3.6%)	
18.5–24.9 kg/m^2^	187/420 (44.5%)	186/393 (47.3%)	
25.0–29.9 kg/m^2^	110/420 (26.2%)	94/393 (23.9%)	
≥ 30 kg/m^2^	104/420 (24.8%)	99/393 (25.2%)	
Marital status (n (%))			0.401
Single	36/407 (8.8%)	26/380 (6.8%)	
Married	188/407 (46.2%)	199/380 (52.5%)	
Common-law spouse	179/407 (44.0%)	153/380 (40.3%)	
Education (n (%))			0.434
Without a high school diploma	38/410 (9.3%)	27/390 (6.9%)	
High school diploma	65/410 (15.9%)	59/390 (15.1%)	
College diploma/trade school	142/410 (34.6%)	128/390 (32.8%)	
University diploma	165/410 (40.2%)	176/390 (45.1%)	
Ethnic or racial background (n (%))			0.074
Caucasian	346/421 (82.2%)	340/392 (86.7%)	
Other	75/421 (17.9%)	52/392 (13.3%)	
Gestational age at first visit (week)^a^	11.1 ± 2.0	11.3 ± 1.68	0.219
Gestation duration^a^	39.2 ± 1.3	39.3 ± 1.6	0.152
First-pregnancy (n (%))	204/422 (48.5%)	204/394 (51.6%)	0.327
Adherence (number of visits)^a^	9.6 ± 1.7	10.4 ± 1.5	< 0.001
History of GD (n (%))	12/422 (2.8%)	9/394 (2.3%)	0.614
History of GHT (n (%))	12/421 (2.9%)	15/393 (3.8%)	0.442
History of depression/anxiety (n (%))	135/422 (32.0%)	114/394 (28.9%)	0.343
Neonatal sex			
Male	206/421(48.9%)	203/389 (52.2%)	
Female	215/421 (51.1%)	186/389 (47.8%)	0.355

*Abbreviations*: *BMI* Body mass index, *GD* Gestational diabetes, *GHT* Gestational hypertension

^a^mean ± standard deviation

### Gestational weight gain

The odds of excessive GWG were 29% lower in the group of women exposed to enhanced care compared with women who were followed during the standard care period (Table 2). For women with pre-pregnancy BMI in the obese category, the intervention was associated with three times higher odds that they complete their pregnancy with adequate weight gain. However, for women with other pre-pregnancy BMI categories, the odds of ending the pregnancy with adequate or excessive GWG were comparable across study groups. In additional analyses, we observed that for women who were underweight at the beginning of their pregnancies, enhanced care was associated with a lower likelihood of completing the pregnancy with an adequate GWG as more of these women ended with a low GWG. In sum, the average GWG was similar between women in the two groups. We ran sensitivity analyses where the models above were repeated with participants excluding those who had GD. Results from these analyses were the same as those described for the full sample above, indicating lower odds of excessive GWG among women exposed to enhanced care compared with women who were followed during the standard care period.

**Table 2:** GWG in the intervention and comparison groups

	Intervention (*n* = 422)	Comparison group (*n* = 394)	*p*-value	unadjusted Odds ratio (95% CI)	adjusted Odds ratio (95% CI)
GWG					
Adequate GWG	156/392 (39.8%)	124/382 (32.5%)	0,093	Reference	Reference
Low GWG	92/392 (23.5%)	95/382 (24.9%)		0.77 (0.53—1.15)	0.71 (0.48—1.04)
Excessive GWG	144/392 (36.7%)	163/382 (42.7%)		0.70 (0.51—0.97)	0.71 (0.49—0.99)
GWG excluding those with GD					
Adequate GWG	153//382(40.1%)	123/370 (33.2%)	0.13	Reference	Reference
Low GWG	88/382 (23.0%)	89/370 (24.1%)		0.80 (0.54—1.16)	0.76 (0.51—1.11)
Excessive GWG	141/382 (36.9%)	158/370 (42.7%)		0.72 (0.52—0.99)	0.71 (0.50—0.99)
Adequate GWG by BMI					
BMI < 18.5	6/17 (35.3%)	9/13 (69.2%)	0.100	0.15 (0.02—0.94)	0.17 (0.02—1.42)
BMI 18.5–24.9 kg/m^2^	84/171 (49.1%)	72/185 (38.9%)	0.102	1.35 (0.83—2.20)	1.35 (0.83—2.22)
BMI 25.0–29.9 kg/m^2^	36/103 (35.0%)	28/91 (30.8%)	0.629	0.92 (0.36—2.38)	0.84 (0.30—2.34)
BMI ≥ 30 kg/m^2^	30/101 (29.7%)	15/93 (16.1%)	0,062	2.67 (1.12—6.37)	3.37 (1,31—8.67)
Excessive GWG by BMI					
BMI < 18.5	2/17 (11.8%)	2/13 (15.4%)	0.100	0.22 (0.02—2.67)	0.23 (0.01—5.63)
BMI 18.5–24.9 kg/m^2^	36/171 (21.1%)	54/185 (29.2%)	0.102	0.77 (0.44—1.36)	0.78 (0.43—1.40)
BMI 25.0–29.9 kg/m^2^	53/103 (51.5%)	53/91 (58.2%)	0.629	0.71 (0.29—1.75)	0.70 (0.27—1.85)
BMI ≥ 30 kg/m^2^	53/101 (52.5%)	54/93 (58.1%)	0.062	1.31 (0.64—2.69)	1.68 (0.74—3.79)
	Intervention (*n* = 422)	Comparison group (*n* = 394)	*p*-value	unadjusted β (95% CI)	adjusted β (95% CI)
Total GWG (kg)^a^	12.3 ± 5.4 (*n* = 394)	12.4 ± 5.4 (*n* = 383)	0,642	0.02 (-0.58—0.95)	0.01 (-0.66—0.78)
Total GWG z-score^a^	-0.02 ± 1.0 ((*n* = 394)	0.02. ± 1.0 ((*n* = 383)	0,642	0.02 (-0.11—0.18)	0.01 (-0.12—0.14)
Total GWG by BMI (kg)^a^					
BMI < 18.5 kg/m^2^	12.1 ± 4.5 (*n* = 17)	14,5 ± 3.4 (*n* = 13)	0,133	0.08 (-0.76—5.42)	0.21 (-1.02—5.35)
BMI 18.5–24.9 kg/m^2^	13.5 ± 4.6 (*n* = 171)	13.8 ± 4.6 (*n* = 185)	0,646	0.01 (-0.74—1.19)	0.08 (-0.83—1.07)
BMI 25.0–29.9 kg/m^2^	12.2 ± 5.2 (*n* = 103)	12.8 ± 5.4 (*n* = 91)	0,447	0.03 (-0.92—2.07)	0.03 (-1.06—2.02)
BMI ≥ 30 kg/m^2^	10.1 ± 6.2 (*n* = 101)	9.1 ± 5.9 (*n* = 93)	0,262	0.07 (-2.71—0.74)	0.10 (-3.07—0.23)

*Abbreviations*: *BMI* Body mass index, *GWG* Gestational weight gain, *CI* Confidence intervals, GWG is adjusted for GD, *BMI* Parity, age, education of the mother & ethnicity

^a^mean ± standard deviation

In the enhanced care group, 3.2% of women developed GHT compared to 11.8% of women who received standard care (Table 3). The odds of developing GHT were therefore 73% lower among women in the group who received enhanced care, in comparison with women in the standard care group. The mean systolic and diastolic BP during pregnancy was higher among women in the standard care group. In particular, the mean systolic BP was higher at the first and second trimester of pregnancy among women in the standard care group, compared to those in the enhanced care group. However, there was no difference between the two study groups with respect to GD. The same was true for mean blood glucose one hour after ingestion of 50 g of glucose, which did not differ between the two groups.

**Table 3:** Maternal and obstetrical outcomes in the intervention and comparison groups

	Intervention (*n* = 422)	Comparison group (*n* = 394)	*p*-value	unadjusted Odds ratio (95% CI)	adjusted Odds ratio (95% CI)
GD (n (%))	13/414 (3.1%)	15/391 (3.8%)	0.59	0.81 (0.38—1.73)	0.48 (0.12—1.94)
Abnormal glycemia one h after ingestion of 50 g of glucose (mmol/L) (n (%))	53/408 (48.2%)	57/382 (51.8%)	0.433	0.85 (0.57—1.27)	0.56 (0.30—1.06)
GHT (n (%))	13/411 (3.2%)	46/389 (11.8%)	< 0.001	0.24 (0.13—0.46)	0.27 (0.14—0.53)
Induction of labor					
GD (n (%))	2/414 (0.5%)	7/391 (1.8%)	0.078	0.27 (0.06—1.29)	0.31 (0.06—1.52)
GHT/preeclampsia n (%)	5/411 (1.2%)	25/389 (6.4%)	< 0.001	0.18 (0.07—0.47)	0.18 (0.07—0.49)
Macrosomia (n (%))	1/422 (0.2%)	2/394 (0.5%)	0.523	0.48 (0.04—5.42)	0.49 (0.04—5.42)
Other (n (%))	15/422 (3.6%)	34/394 (8.6%)	0.002	0.39 (0.21—0.73)	0.41 (0.22—0.77)
Induction of labor or C-section due to GD, GHT/preeclampsia, obesity, or macrosomia (n (%))	7/403 (1.7%)	35/387 (9.0%)	< 0.001	0.18 (0.08—0.41)	0.19 (0.08—0.43)
Type of delivery					
Vaginal (n (%))	297/422 (70.4%)	277/393 (70.5%)	0.974	1.01 (0.74—1.36)	1.01 (0.74—1.38)
C-section (n (%))	125/422 (29.6%)	116/393 (29.6%)			
*Perineal tears*					
Perineum intact (n (%))	58/293 (19.8%)	41/270 (15.2%)	0.151	1.38 (0.89—2.14)	1.42 (0.89—2.25)
1^st^ degree (n (%))	86/293 (29.4%)	66/270 (24.4%)	0.19	1.28 (0.88—1.87)	1.28 (0.88—1.87)
2^nd^ degree (n (%))	136/293 (46.4%)	143/270 (53.0%)	0.121	0.76 (0.55—1.07)	0.77 (0.55—1.07)
3^rd^ degree (n (%))	13/293 (4.4%)	19/270 (7.0%)	0.183	0.61 (0.29—1.27)	0.62 (0.30—1.29)
Episiotomy (n (%))	28/293 (9.6%)	35/270 (13.0%)	0.2	0.70 (0.41—1.20)	0.71 (0.42—1.20)
	Intervention (*n* = 422)	Comparison group (*n* = 394)	*p*-value	unadjusted β (95% CI)	adjusted β (95% CI)
Glycemia one hr after ingestion of 50 g of glucose (mmol/L)^a^	5,9 ± 1.6 (*n* = 408)	6,2 ± 1.7 (*n* = 382)	0.125	0.03 (-0.05—0.41)	0.13 (0.04—0.64)
Systolic BP (mmHg)^a^	108.3 ± 8.7 (*n* = 422)	110.1 ± 9.6 (*n* = 394)	0.005	0.10 (0.06—0.33)	0.09 (0.07—0.31)
Diastolic BP (mmHg)^a^	63.1 ± 5.5 (*n* = 422)	63.9 ± 6.3 (*n* = 394)	0.036	0.07 (0.06—1.67)	0.06 (0.04—1.50)
First trimester					
Systolic BP (mmHg)^a^	107.2 ± 12.1 (*n* = 373)	109.1 ± 13.4 (*n* = 368)	0.048	0.07 (0.02—0.29)	0.08 (0.02—0.30)
Diastolic BP (mmHg)^a^	62,8 ± 7.8 (*n* = 373)	62.5 ± 8.6 (*n* = 368)	0.592	-0.02 (-0.18—0.11)	-0.02 (-0.17—0.11)
Second trimester					
Systolic BP (mmHg)^a^	106.6 ± 9.6 (*n* = 420)	108.7 ± 10.2 (*n* = 394)	0.003	0.11 (0.07—0.35)	0.10 (0.08—0.32)
Diastolic BP (mmHg)^a^	61.4 ± 6.1 (*n* = 420)	61.5 ± 6.4 (*n* = 394)	0.828	0.01 (-0.12—0.15)	0.01 (-0.13—0.13)
Third trimester					
Systolic BP (mmHg)^a^	109.9 ± 9.5 (*n* = 421)	111.2 ± 10.6 (*n* = 392)	0.075	0.06 (-0.02—0.26)	0.06 (-0.01—0.24)
Diastolic BP (mmHg)^a^	64.5 ± 6.5 (*n* = 421)	65.6 ± 7.4 (*n* = 392)	0.026	0.08 (0.02—0.30)	0.07 (0.11—0.27)

*Abbreviations*: *GHT* Gestational hypertension, *BP* Blood pressure, *GD* Gestational diabetes, *CI* Confidence intervals, GD is adjusted for BMI, parity, history of GD and macrosomia, age & education of the mother, GHT is adjusted for GD, *BMI* Parity, history of GHT, age & education of the mother, Induction of labor, type of delivery and perineal tears adjusted for BMI, age, and parity

^a^mean ± standard deviation

Among the other outcomes studied, the odds of labor induction or C-section due to pregnancy complications were 81% lower in the group that received enhanced care compared to the group that received standard care. Specifically, 1.2% of the women in the enhanced care group had an induction of labor and delivery due to GHT and/or preeclampsia compared to 6.4% of the women in the comparison group. There was also a difference between the two groups in the odds of induction of labor and delivery for other reasons (oligohydramnios, post-term, etc.), but induction of labor and delivery due to GD or macrosomia did not differ between the groups. In addition, although the distribution of methods of delivery did not differ between the study groups, perineal tears and episiotomies were more common among women in the standard care group compared to women in the enhanced care group.

### Neonatal outcomes

The average birth weight of newborns was significantly lower among newborns in the enhanced care group compared to those in the standard care group (Table 4). The odds of fetal macrosomia were 44% lower among newborns in the enhanced care group compared with newborns in the standard care group. Similarly, the odds of LGA were 40% lower among newborns in the enhanced care group. However, there were no statistically significant differences between the two study groups regarding premature birth, shoulder dystocia, and Apgar score at one and five minutes of life.

**Table 4:** Neonatal outcomes in the intervention and comparison groups

	Intervention (*n* = 422)	Comparison group (*n* = 393)	*p*-value	unadjusted Odds ratio (95% CI)	adjusted Odds ratio (95% CI)
Sex					
Female (n (%))	215/421 (51.1%)	186/389 (47.8%)	0.355	0.87 (0.66—1,15)	0.89 (0.67—1.17)
Male (n (%))	206/421 (48.9%)	203/389 (52.2%)			
Macrosomia (> 4 kg) (n (%))	30/396 (7.6%)	46/380 (12.1%)	0.034	0.60 (0.37—0.97)	0.56 (0.34—0.93)
Large for gestational age (n (%))	31/421 (7.4%)	45/392 (11.5%)	0.044	0.61 (0.38—0.99)	0.60 (0.36—0.99)
Premature birth (n (%))	26/422 (6.2%)	14/393 (3.6%)	0.085	1.78 (0.92—3.47)	1.81 (0.93—3.53)
*Shoulder dystocia* (n (%))	26/293 (8.9%)	34/270 (12.6%)	0.153	0.68 (0.40—1.16)	0.65 (0.38—1.12)
	Intervention (*n* = 422)	Comparison group (*n* = 393)	*p*-value	unadjusted β (95% CI)	adjusted β (95% CI)
Weight at birth (g)	3400.0 ± 450.8 (*n* = 396)	3470.1 ± 445.8 (*n* = 380)	0.03	0.07 (0.01—0.30)	0.08 (0.03—0.30)
Apgar score at 1 min (n (%))	8.6 ± 1.3 (*n* = 422)	8.4 ± 1.5 (*n* = 392)	0.196	0.02 (-0.32—0.06)	0.02 (-0.30—0.08)
Apgar score at 5 min (n (%))	8.9 ± 0.5 (*n* = 422)	8.8 ± 0.7 (*n* = 392)	0.112	0.03 (-0.16—0.02)	0.01 (-0.15—0.02)

Birth weight, macrosomia and LGA are adjusted for parity, age & education of the mother, and interaction between GD, BMI, and GWG

^a^mean ± standard deviation

## Discussion

In this study, a simple intervention combining PA education from a nurse and prescription of PA from a physician during pregnancy follow-up was associated with lower odds of excessive GWG, GHT, fetal macrosomia, LGA and onset of labor and delivery due to GHT and/or preeclampsia.

In the present study, a combination of education and PA prescription was associated with a six-percentage point lower proportion of women exceeding the recommended GWG. Previous studies that had investigated the effects of personalized GWG education [59] or a combination of personalized GWG education and PA prescription in maternal care [7] had not found significant effects. It is possible that our study differs from others by having adopted a mode of operation that was designed to be easily integrated into the setting and practices of an obstetric clinic without requiring additional resources or considerable time. In addition, the combination of actions by a nurse and a physician may also have contributed to improve the potential of the intervention. A review of systematic reviews and meta-analyses showed that physician advice is effective in increasing PA in the short term [34]. However, PA prescriptions are rarely used as physicians identify time constraints as a barrier to their implementation [60, 61]. In the current study, nurses provided education on PA and assessed women’s intention to change their PA level, which allowed physicians to rapidly complete their PA prescriptions. Physician interventions are thought to be effective because they are perceived by the population as the most credible source of health information [39, 62]. In addition, PA prescriptions provide a concrete reinforcement of the importance of the recommended action [39, 41, 63]. Similarly, it is possible that women became more sensitised to the importance of PA as they noted the complementary actions of the two health professionals. Empirical evidence supports this as it is commonly reported that interprofessional collaborations lead to more effective care and provide better clinical health outcomes for patients [64, 65]. In particular, a systematic review of randomized trials showed that collaborations between nurses and physicians have a positive effect on patient health [64]. This is also consistent with findings indicating that one-on-one PA education offered to pregnant women by nurses and reinforced by physicians encourages greater adherence to PA recommendations [66].

Our results are similar to the results of randomized trials where lifestyle interventions during pregnancy were associated with lower GWG [67]. However, to our knowledge, our intervention is the only one to suggest an association with lower odds of GHT and lower mean systolic and diastolic BP [55, 59, 68]. Our results also differ from studies in which lifestyle interventions did not relate to differences in newborn birth weight or macrosomia [7, 55, 69–71]. Our results are nevertheless similar to those of others who documented a reduction in risk of macrosomia and LGA following a lifestyle intervention [59, 70]. It is also relevant to mention that although the intervention did not aim to improve all maternal and fetal outcomes, such as type of delivery, perineal tears, episiotomy, prematurity, shoulder dystocia, and Apgar score at 1 and 5 min, improvements in several outcomes were noted.

Beyond the practicability of the intervention, the power of prescriptions and the advantages of interprofessional collaborations, it is possible that gains in GWG awareness through a personalized weight gain chart positively influenced outcomes in the intervention group. It has been previously shown that pregnant women who follow weight gain based on pre-pregnancy BMI are three times more likely to achieve recommended pregnancy weights [72]. It is also conceivable that tailoring PA prescriptions based on behavioral stages of change has had the beneficial effect of improving some maternal and fetal outcomes. Systematic reviews [45, 73] have demonstrated that stages of behavior change are directly related to PA in adults [45] and other reviews have documented that interventions based on this model are generally effective in changing PA behavior [46–48, 73, 74].

Despite its association with several improvements in maternal and fetal health outcomes, the intervention tested in the present study was not associated with a lower prevalence of GD. Lifestyle interventions have had mixed results on GD with some showing no effects [59, 68, 70] and others being associated with a reduction in GD [55, 75]. In general, interventions that succeed in preventing GD focused on diet [16, 55, 75]. Our study did not focus on diet given that the obstetric clinic where the study took place already provided detailed education about diet during pregnancy. Thus, it is possible that the potential for further improvement was reduced given that the clinic’s standard of care already included an intervention targeting one of the key predictors of GD. It is also possible that the development of GD was associated with pre-pregnancy dysglycemia or a metabolic maladjustment developed early in pregnancy and against which PA or dietary interventions would have limited effects [76]. This would be in line with results from the LIFE-Moms consortium, which demonstrated that multi-lifestyle interventions can have beneficial effects on GWG among women with overweight and obese pre-pregnancy weight without impacting GD [77].

It is also noteworthy that women in the standard care group in this study attended more prenatal care visits than women in the intervention group. It is possible that this was related to the emergence of the COVID-19 pandemic, which overlapped only with the enhanced care period. Given timing of study periods, for most participants who would have been affected by pandemic-related restrictions, it is their later-pregnancy related visits that would have been reduced. This, combined with previous observations that it is early-pregnancy interventions that have the most beneficial effects [78], may explain why we could still observe better maternal and fetal outcomes among the intervention group.

A strength of this study is that we succeeded in recruiting 95% of women eligible, which possibly reduced the risk of selection bias and improved potential that our results could be generalizable to other settings. However, women who consented to participate in the intervention group might still be different from those who were in the comparison group, which constitutes a limitation of the study. Also, whereas randomized control trials represent the gold standard design for assessing the effectiveness of interventions [79, 80], the research team opted not to use this design out of a sense of fairness, in order to allow all women to benefit from the preventive intervention that was anticipated to be favorable for their health [81]. Nevertheless, the study design used adapts well to the constraints of natural environments since there is no indication that factors other than the intervention may have had a significant influence in differentiating the two time periods under study [9]. One exception to this is the occurrence of the COVID-19 pandemic which overlapped only with part of the enhanced care period. Although other studies suggested that the pandemic was associated with a worsening of physical activity levels and other health related behaviors at a population level [82, 83]. It is possible that other factors related to the pandemic were associated with the better outcomes observed among participants in the enhanced care group. Further, participants in this study were from a single hospital, which may reduce the generalizability of the results to other settings. Data collected on pre-pregnancy weight were self-reported, which may reduce the accuracy of this information. For this variable, most studies have noted a slight underestimation of self-reported values [84]. However, because both groups are subject to this same underestimation, the possibility of social desirability bias should not impact the overall findings [84]. GWG was calculated as the difference between the last recorded pregnancy weight and the maternal weight measured during the first prenatal visit. It is possible that the GWG was underestimated by not considering the WG in the early weeks of pregnancy. However, the literature suggests that this limitation is minimal since the majority of GWG occurs in the second or third trimester of pregnancy [56]. Furthermore, although the proportion of women who developed pregnancies using assisted reproduction technology was likely similar between groups, we did not collect information on this variable, which is known to be associated with a higher risk of adverse obstetric outcomes [85]. To ensure fidelity of intervention implementation throughout the duration of the study, intervention evaluation tools were used, numerous follow-ups were conducted with the care team, and training was provided. Despite these control measures, it is not possible to control whether the intervention was consistently delivered, and we have no information on whether participants were exposed to the intervention as planned. It is also possible that other perinatal factors, which we are not aware of, differentially influenced study outcomes across study groups.

## Conclusion

This study suggests that using a combination of nurse education and physician prescription of PA according to the transtheoretical model in a routine prenatal care setting is associated with better maternal and fetal health outcomes. In this study, the intervention was most strongly associated with lower odds of excessive GWG, GHT, fetal macrosomia, induction of labor, or having a C-section because of obesity, GHT, GD, and macrosomia.

## Data Availability

The data that support the findings of this study are available on request from the corresponding author [L. S.]. The data are not publicly available due to “them containing information that could compromise research participant privacy/consent”.

## Abbreviations

BMI: Body mass index
BP: Blood pressure
CI: Confidence interval
DGLDUHC: Dr. Georges-L.-Dumont University Hospital Centre
GD: Gestational diabetes
GHT: Gestational hypertension
GWG: Gestational weight gain
IOM: Institute of Medicine
OR: Odds ratio
PA: Physical activity
